# Mesencephalic Astrocyte-Derived Neurotrophic Factor (MANF): An Emerging Therapeutic Target for Neurodegenerative Disorders

**DOI:** 10.3390/cells12071032

**Published:** 2023-03-28

**Authors:** Bhadrapriya Sivakumar, Anand Krishnan

**Affiliations:** 1 Department of Anatomy, Physiology, and Pharmacology, College of Medicine, University of Saskatchewan, Saskatoon, SK S7N 5E5, Canada; 2 Cameco MS Neuroscience Research Centre (CMSNRC), Saskatoon, SK S7K 0M7, Canada

**Keywords:** MANF, ER stress, UPR, neuroprotection, nerve regeneration, apoptosis

## Abstract

Mesencephalic astrocyte-derived neurotrophic factor (MANF) is a member of the new family of neurotrophic factors (NTFs) with a unique structure and functions compared to other conventionally known NTFs. MANF is broadly expressed in developing and mature tissues, including the central nervous system and peripheral nervous system tissues. Growing research demonstrated that MANF protects neurons from endoplasmic reticulum (ER) stress-associated complications by restoring ER homeostasis and regulating unfolded protein response. This review discusses MANF signaling in neurodegenerative conditions with specific emphasis given to its overall effect and mechanisms of action in experimental models of Parkinson’s disease, Alzheimer’s disease, and stroke. Additional perspectives on its potential unexplored roles in other neurodegenerative conditions are also given.

## 1. Introduction

Mesencephalic astrocyte-derived neurotrophic factor (MANF) is a neurotrophic factor that is structurally different from other conventionally known neurotrophic factors, such as nerve growth factor (NGF) and brain-derived neurotrophic factor (BDNF). The presence of a saposin-like domain at the N-terminal and SAP domain at the C-terminal of MANF pose this structural difference [1]. Accumulating studies showed that MANF promotes the survival of dopaminergic neurons, especially by modulating endoplasmic reticulum (ER) stress and unfolded protein response (UPR) [2,3]. MANF was initially discovered by Petrova et al. in rat mesencephalic type 1 astrocyte culture media. Initial sequencing studies showed its 100% homology with human arginine-rich protein (hARP) [2]. However, the N-terminal arginine-rich sequence in the hARP is not present in MANF [2]. In other words, hARP is MANF coupled with an N-terminal arginine-rich sequence.

MANF is evolutionarily conserved in both vertebrates and invertebrates [2]. Human MANF shows approximately 50% sequence similarity with Drosophila and *C. elegans* MANF [4]. MANF is expressed in both neuronal and non-neuronal tissues. For example, MANF is shown to express in the brain tissues of *C. elegans*, Drosophila, zebrafish, and rodents [5,6,7,8]. Similarly, MANF is expressed in non-neuronal tissues such as the heart, kidney, salivary gland, and pancreas [9,10,11,12]. In rat brain tissue, MANF is expressed in the cerebral cortex, hippocampus (CA1-CA3 and dentate gyrus), substantia nigra (SN), and striatum, while in Zebrafish it is expressed in the preoptic, ventral thalamic, pretectal, dorsal thalamic, and hypothalamic regions [7,8].

Cerebral dopamine neurotrophic factor (CDNF) is another neurotrophic factor that is structurally and functionally quite similar to MANF. The CDNF consists of 161 amino acid residues. Human CDNF has approximately 45–59% sequence similarity to the MANF present in various species [4]. However, critical modifications, such as the presence of lysine residue K112 in MANF, instead of the leucine residue in CDNF, pose differences in their biological properties, especially their cell surface binding affinity [13]. Additionally, a recent study by Pakarinen et al. showed that the functions of MANF cannot be replaced by CDNF in mouse brain and pancreatic tissues, suggesting that MANF and CDNF possess unique functions [14].

## 2. Structure-Activity Relationship of MANF

**Figure 1:**
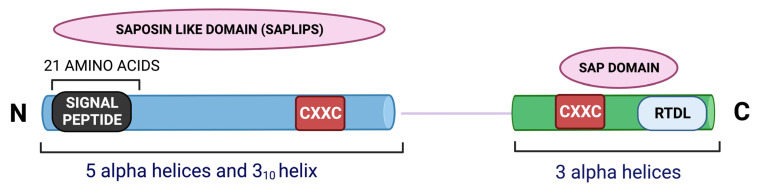
An illustration of the structure of MANF.

MANF is a small protein consisting of 179 amino acids [2]. It is comprised of two domains, the N terminal and the C terminal, which are interconnected with a short linker. A simplified structure of MANF is illustrated in Figure 1. The 21 amino acids at the N-terminal region of MANF serve as the signal sequence [2]. NMR studies showed that the N terminal domain of MANF is homologous to saposin-like proteins (SAPLIPs) and contains five α-helices and a 3_10_-helix configured into a closed globular structure through three disulfide bridges [1]. SAPLIPs are a small class of cysteine-rich proteins that interact with membrane lipids, such as lipid sulfatide [13]. Studies in *C. elegans* and mammalian cardiomyocytes demonstrated that MANF binds to lipid sulfatide on the cell surface and this binding enables its cellular intake [13]. Bai et al. observed that the addition of exogenous sulfatide to the culture media enhances the uptake of MANF in HEK293T cells [13]. The authors of the study also demonstrated that the lysine residue K112 in MANF is critical for its sulfatide binding, and hence, CDNF may not possess this property. The sulfatide binding of MANF was also shown to facilitate its cytoprotective effect in *C. elegans* and mammalian cardiomyocytes [13].

The C terminal of MANF contains three helices—a loose α-helix and two additional α-helices in parallel orientation forming a helix-loop-helix orientation. This is homologous to the SAP domain (SAF-A/B, Acinus and PIAS) of Ku70 protein as resolved by NMR spectroscopy [1]. It is known that the SAP domain of Ku70 serves as an anti-apoptotic domain and inhibits the pro-apoptotic bax [15]. The C terminal of MANF was found to protect superior cervical ganglion (SCG) neurons from apoptosis in a bax dependent manner, substantiating the functional similarity of the C terminal end of MANF and SAP domain of Ku70 [1]. The SAP-like domain also facilitates MANF’s interaction with the NFκB subunit p65. This interaction negatively regulates NFκB signaling during ER stress and inflammation [16]. Hence, the C-terminal or the SAP-like domain of MANF is as critical as its N-terminal region for mediating cytoprotective actions.

MANF has eight cysteine and two CXXC motifs, one per domain. The CXXC motif at the C terminal contains an internal disulfide bond between two cysteines [17]. Other proteins with CXXC motifs found abundantly in the ER include protein disulfide isomerase (PDI) and reductase [17]. However, MANF does not show any PDI activity [18]. Strikingly, a mutation in the CXXC motif was shown to abolish MANF’s neuroprotective property. For example, Mätlik et al. observed that the ability of MANF to protect SCG neurons from the cytotoxic drug etoposide was abolished when cysteine 151 of CXXC was mutated to serine [19].

MANF is a secretory protein primarily localized to the luminal ER [19,20]. The C-terminal of MANF contains an RTDL motif that is recognized by KDEL receptors and enables its ER-Golgi retention [20]. The ER chaperone binding immunoglobulin protein/glucose-regulated protein 78 (BiP/GRP78) is also a substrate for the KDEL receptor [21]. During ER stress, the expression of both GRP78 and MANF increases, and they might compete with each other for the KDEL receptor, resulting in the retention of GRP78 and secretion of MANF [22].

Although MANF was shown to protect neurons, whether the full-length or specific residues of MANF are critical for its neuroprotective functions was an interesting question addressed by several researchers. In Drosophila, the full-length MANF was shown to be necessary for its neuroprotective activity as the removal of either the N-terminal or the C-terminal or both failed to rescue dopaminergic axons, and the fly, from lethality [23]. Similarly, studies in *C. elegans* showed that the removal of MANF’s three conserved cysteines and half of the N terminal domain induces behavioral defects. For example, an electrotaxis assay showed that the swimming speed of adult *C. elegans* expressing this MANF-mutant was reduced by over 30% [5]. MANF was also shown to protect neurons from ER stress-induced damage in Parkinson’s disease (PD), Alzheimer’s disease (AD), and stroke. The following section of the review will discuss the mechanisms of MANF’s neuroprotective and nerve-regenerative actions [24,25,26,27].

## 3. The Crosstalk between ER Stress, UPR, and MANF

Reduction in calcium levels, viral infections, pharmacological perturbations, abnormal protein expression, and altered protein glycosylation can cause aggregation of misfolded or unfolded proteins in the ER, leading to ER stress. The ER stress, if not controlled, induces cellular apoptosis [28]. Chronic ER stress underlies several pathological disorders including diabetes, PD, AD, and glomerular and tubular kidney diseases [25,29,30,31]. In these pathological states, intrinsic cellular mechanisms attempt to counteract ER stress by activating UPR signaling. The activation of UPR, in turn, limits new protein translation, facilitates protein folding, and induces degradation of misfolded proteins to ensure protein homeostasis [32]. UPR is regulated by three ER-resident transducers: the endoribonuclease inositol requiring enzyme 1 alpha (IRE1α), protein kinase RNA-like endoplasmic reticulum kinase (PERK), and activating transcription factor 6 (ATF6) [32]. They have three domains: an ER luminal domain, a transmembrane domain, and a cytosolic domain. At steady-state, the ER chaperone GRP78 forms a complex with the ER transducers keeping them inactive. During ER stress, the GRP78 dissociates from the complex and binds to misfolded proteins in the ER lumen resulting in the release of the ER transducers [33].

The sequential events of UPR transduction are reviewed in detail in [32,34]. Following the release from the GRP78 complex, the cytosolic portion of IRE1α undergoes autophosphorylation, resulting in the activation of its endonuclease activity. The IRE1α then splices the X-box binding protein 1 (XBP1) mRNA. The spliced XBP1 (sXBP1) is a transcriptional activator. It upregulates UPR target genes and facilitates protein folding, protein degradation, and protein transport, thus reducing the burden of misfolded proteins in the ER [34]. IRE1α also cleaves mRNAs and miRNAs for reducing the overall protein burden in the ER [32]. PERK also possesses kinase activity and phosphorylates eukaryotic translational initiation factor 2α (eIF2α). The eIF2α puts a brake on the overall protein synthesis but selectively spares the transcription factor ATF4. The ATF4, in turn, translocates to the nucleus and facilitates the transcription of the molecules that regulate UPR [32,34]. PERK also activates C/EBP homologous protein (CHOP), which, in turn, mediates cellular apoptosis [35]. The third UPR transducer ATF6 translocates to the Golgi, where it is cleaved by the Site-1-Protease (S1P) and Site-2-Protease (S2P). It then releases the transcription factor bZIP, which transits to the nucleus and upregulates UPR genes [32,34].

Several studies demonstrated that ER stress induces MANF upregulation. ER stress in pathological conditions, such as rheumatoid arthritis, systemic lupus erythematosus, glomerular disease, liver damage, and multiple myeloma was shown to induce MANF upregulation [16,36,37,38]. Similarly, cell lines treated with the ER stressors, such as thapsigargin, tunicamycin, and lactacystin, also showed MANF upregulation, indicating its critical involvement in ER-stress-related signaling [39,40]. MANF was also shown to overexpress in cortical neurons following ischemia-induced ER stress [40]. Current evidence, in general, indicates that MANF offers protection against ER stress-induced cell death [39,40]. Substantiating this argument, deficiency of MANF was shown to worsen ER stress and neurotoxicity. For example, Wang et al. showed that ethanol induces neurotoxicity in mouse frontal cortex, thalamus, cerebellum, and hippocampus. 4-PBA, a chemical chaperone and inhibitor of ER stress, abolished neuronal apoptosis in this setting, showing the involvement of ER stress in ethanol-induced neurotoxicity [41]. Strikingly, MANF deficiency worsened ethanol-induced ER stress and neurotoxicity in mice, indicating that MANF supplementation may protect animals from ER-stress-mediated neurotoxicity [41].

**Figure 2:**
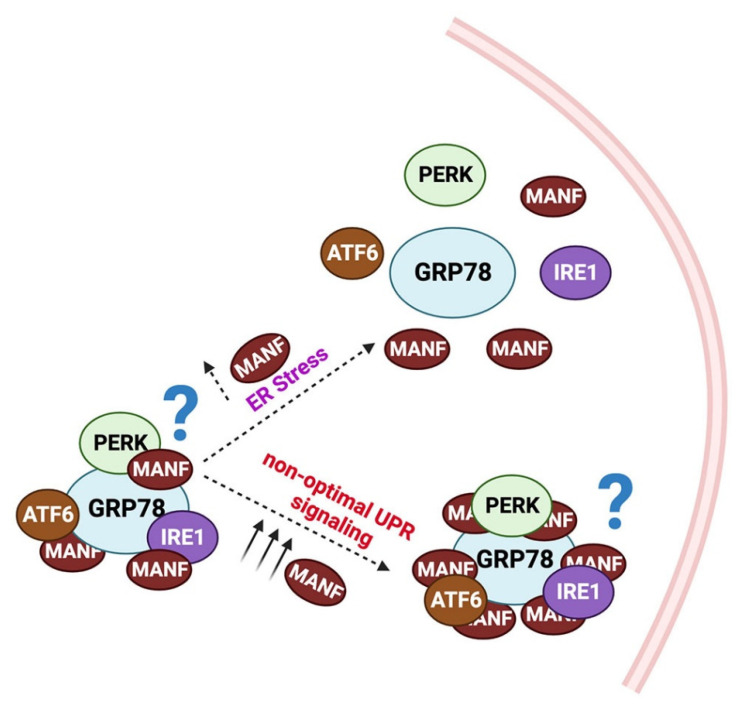
Potential mechanism for MANF-dependent UPR regulation: In the steady state, MANF binds to GRP78. The MANF-GRP78 interaction may stabilize the GRP78/PERK/IRE1α/ATF6 complex. During ER stress, due to high-affinity binding between GRP78 and KDEL receptors, MANF releases from the complex, and hence, the UPR transducers PERK/IRE1α/ATF6. However, in uncontrolled UPR, increased MANF levels re-establish the GRP78/ PERK/IRE1α/ATF6 complex and suppress UPR.

Although the exact mechanism by which MANF counteracts ER stress is currently under investigation, several potential mechanisms are postulated and partially understood. One possibility is the facilitation of protein folding. It is known that the CXXC motif at the C terminal end of MANF catalyzes the formation of intramolecular disulfide bonds [17]. Therefore, it is speculated that MANF may facilitate the formation of disulfide and cysteine bonds in misfolded proteins, and thus, may promote protein folding [42]. Another potential mechanism may be a MANF-induced stabilization of the complex of GRP78 and the UPR transducers PERK, IRE1α, and ATF6. Yan et al. demonstrated that MANF binds to the ADP-bound GRP78 through its C-terminal SAP domain, while Glembotski et al. demonstrated that calcium is required for the formation of the GRP78-MANF complex [22,43]. The authors showed a 60% reduction in the MANF-GRP78 complex following the inhibition of sarco/endoplasmic reticulum calcium ATPase (SERCA) in Hela cells [22]. Regardless, the interaction of MANF with GRP78 may inhibit ATP binding to, and ADP release from GRP78. Substantiating this view, the SAP domain of MANF was shown to inhibit ADP release from Hsp70, indicating its potential to serve as a nucleotide exchange inhibitor [43]. Strikingly, lower levels of complex between GRP78 and α1 antitrypsin were found in MANF knockdown cells, suggesting that MANF may promote the binding of GRP78 with its partners, especially UPR transducers to keep them inactive [43]. However, additional studies are required to substantiate this view. The potential mechanisms that may contribute to MANF-dependent UPR regulation is given in Figure 2.

## 4. MANF Signaling in Parkinson’s Disease (PD)

PD is a progressive neurodegenerating disorder characterized by resting tremors, bradykinesia, postural abnormalities, and muscle rigidity. PD is also characterized by non-motor symptoms such as cognitive impairments [44]. The locomotor defects in PD occur primarily due to the degeneration of dopamine (DA) neurons [45]. Current PD therapies target the restoration of DA signaling by supplementing synthetic DA analogues and preventing DA degradation. No therapies have been developed so far aimed at preventing DA neuron degeneration.

MANF was initially shown to promote the survival of DA neurons in Drosophila, indicating that it may be a potential therapeutic candidate for PD [2]. Palgi et al. later found that Drosophila zygotes lacking MANF degenerate dopaminergic axons, suggesting that MANF is indeed essential for DA neuron homeostasis [6]. Additionally, the authors demonstrated that flies lacking MANF can be rescued from lethality by the introduction of mammalian MANF, further indicating the therapeutic utility of MANF [6]. MANF was also shown to be essential for the survival of DA neurons in *C. elegans*. MANF deficiency resulted in the loss of one-third of DA neurons in *C. elegans,* while exogenous supplementation of MANF reduced such neuron loss [5]. Aggregation of α-synuclein is a pathological feature of PD, and hence, α-synuclein-based PD models are widely used for PD research. A study by Zhang et al. showed that MANF overexpression delayed neurodegeneration in an α-synuclein-based PD model in *C. elegans*. MANF also restored DA levels and suppressed locomotor defects in this model [46].

The neuroprotective roles for MANF were demonstrated in rodent PD models as well. Liu et al. showed that MANF improved motor behaviors in 1-methyl-4-phenyl-1,2,3,6-tetrahydropyridine (MPTP) induced PD model in mice [30]. Levels of DA and its metabolites dihydroxyphenylacetic (DOPAC) and homovanillic acid (HVA) were reduced in mice after MPTP treatment, while MANF supplementation increased their levels [30]. Similarly, a combination of CDNF and MANF was shown to improve the functions of the nigrostriatal system and prevented the loss of DA neurons in a 6-OHDA induced lesion model in rat [47]. Additionally, Hao et al. showed that the supplementation of MANF to the striatum of rat PD models elicits long-term neuroprotective and neuro-regenerative effects in DA neurons and improves behavioral outcomes [48]. Interestingly, a recent study showed that MANF facilitates the neuroprotective effect of the natural compound dendrobine in PD models. For example, dendrobine was shown to reduce MPTP-induced cytotoxicity in SH-SY5Y cells and rat primary midbrain neurons by increasing the expression of MANF. Knockdown of MANF attenuated the ER-stress-relieving effect of dendrobine, substantiating the role of MANF in promoting the neuroprotective actions of dendrobine [49].

Although the above-mentioned studies provided a strong rationale for clinical studies to examine the therapeutic effect of MANF in PD, such studies are lacking so far. A study by Galli et al. demonstrated that MANF levels were substantially increased in PD patients, providing additional rationale for testing MANF in PD patients [50]. The upregulation of MANF in PD patients also indicates the potential of MANF to serve as a maker for the early diagnosis of PD [50].

## 5. MANF Signaling in Alzheimer’s Disease (AD)

AD is an age-related, progressive, and clinically incurable neurodegenerative disorder characterized by dementia. The main pathological features of AD include the presence of amyloid β (Aβ) plaques and neurofibrillary tangles (NFT). The Aβ plaques are neurotoxic [25]. Accumulation of Aβ plaques induces ER stress in neurons, followed by heightened UPR signaling and neuron death. Xu et al. showed that MANF rescues SH-SY5Y cells from Aβ-induced toxicity by modulating UPR signaling [25]. The authors found increased expression of the UPR mediator CHOP, active caspase 3, and TUNEL positivity in Aβ treated MANF knockdown SH-SY5Y cells, indicating a worsening of Aβ mediated UPR and neurotoxicity in MANF deficient conditions. Interestingly, the knockdown of basal levels of MANF also showed an increase in CHOP levels and TUNEL positivity in SH-SY5Y cells, indicating that MANF is also indispensable for neuron homeostasis [25]. Xu et al. also observed increased levels of MANF in neurons in the hippocampus and cortex of the 6-month-old APP/PS1 double transgenic AD mouse model [25]. The MANF overexpression in this model is accompanied by decreased levels of the UPR transducers GRP78, ATF6, spliced-XBP1, CHOP, and p-IRE1, suggesting that MANF may suppress deregulated UPR in AD [25].

Similar to PD, clinical studies addressing the therapeutic utility of MANF in AD are sparse. Liu et al. studied the distribution of MANF in the inferior temporal gyrus of the cortex (ITGC) of AD patients [51]. They found a partial co-localization of MANF and GRP78 in the ER. Additionally, they noted a higher number of MANF-positive neurons in the ITGC of pre-AD and AD brains compared to non-AD brain samples, showing that MANF is upregulated in AD and may serve as a marker for AD [51].

## 6. MANF Signaling in Stroke

Stroke is one of the leading causes of disability in humans worldwide [18]. It is caused by local thrombosis or hemorrhage, leading to a lack of blood supply to the corresponding brain region, resulting in neuron death [52]. Current stroke management includes endovascular thrombectomy or thrombolytic tissue plasminogen activator (tPA) therapy [53]. The protective role for MANF in stroke has been studied by several groups. Li et al. found that MANF levels are upregulated after the induction of subarachnoid hemorrhage (SAH) in rats [54]. Interestingly, additional supplementation of MANF improved the neuro-deficits in SAH animals. Evan’s blue dye shows a leak in rat SAH experimental models, indicating a breach in blood-brain barrier (BBB) integrity. Interestingly, in rat SAH models supplemented with MANF, the dye leak was comparatively low, indicating that MANF protects BBB. MANF treatment also reduced brain edema and improved falling latency and sensorimotor functions in SAH rats, suggesting that it may improve stroke outcomes. The MANF-mediated protection of BBB was associated with a reduction in matrix metalloprotease-9 (MMP-9) levels, indicating that MANF may modulate MMP-9 activity for offering BBB protection [54]. A study by Mätlik et al. also showed functional improvement in stroke models following MANF treatment [55]. The authors showed that AAV7-mediated supplementation of hMANF improved neurological indices in rat stroke models 14 days after stroke surgery as assessed by Bederson’s neurological score test (BNST) and elevated body swing test (EBST). The rats administered with AAV7-MANF also exhibited a faster reversal of injury-induced behavioral deficits in EBST and cylinder tests compared to control animals. The authors showed that the ischemic injury in rats upregulated MANF in neurons and glial cells. Importantly, the deletion of MANF increased the infarct volume in these rats, substantiating the neuroprotective role of MANF in stroke [55].

In addition to its direct actions, MANF was also shown to promote the neuroprotective actions of potential therapeutic agents in stroke. A study by Belayev et al. showed that MANF facilitated the neuroprotective effect of docosahexaenoic acid (DHA) in a stroke model [56]. The authors showed that DHA supplementation improved neurological scores and behavioral outcomes in rats that had undergone mid-cerebral artery occlusion surgery. Interestingly, these functional improvements were accompanied by the induction of MANF in the brain regions, such as the ipsilateral penumbra, subventricular zone, and dentate gyrus in this model, substantiating the involvement of MANF in facilitating the neuroprotective effect of DHA [56].

MANF was also shown to increase blood flow in the brain regions of stroke models. Using laser doppler flowmetry, Gao et al. showed that the blood flow of the middle cerebral artery (MCA) is improved after MANF treatment in a rat stroke model, indicating that post-stroke administration of MANF may improve blood flow in the peri-infarct area [57]. The angiogenic markers, CD34, VEGF, and Ang1 were also upregulated in the peri-infarct cerebral cortex following MANF treatment, indicating that MANF may promote angiogenesis, which is a key process involved with intrinsic brain repair [57]. However, the exact mechanism by which MANF promotes angiogenesis needs additional investigation.

A summary of MANF’s actions in PD, AD, and stroke is depicted in Table 1.

## 7. Mechanisms of MANF’s Actions Independent of the ER Stress-UPR Axis

While several studies suggest MANF’s ability to suppress UPR signaling as the major mechanism for its neuroprotective effect, additional mechanisms also contribute to its protective actions. PI3K/Akt signaling is well known to promote neuron survival and growth [58]. A study by Airavaara et al. showed that MANF elicits its neuroprotective actions by activating P13K/Akt/mTOR signaling [26]. Interestingly, in another study, Hao et al. noted that MANF treatment did not modify the expression of critical UPR transducers in PD rats [48]. The authors speculated that MANF may protect neurons through other mechanisms, such as activation of P13K/Akt/mTOR signaling. Supporting this view, they found induction of p-Akt and p-mTOR in MANF-treated animals compared to control. The authors also found that the P13K inhibitor wortmannin attenuated MANF-mediated viability of SH-SY5Y cells, substantiating that MANF offers neuroprotection via P13K/Akt/mTOR signaling [48]. Similarly, Zhang et al. showed that MANF induced the expression and nuclear translocation of the master regulator of antioxidant genes, the nuclear factor erythroid-2-related factor 2 (Nrf2) [59]. The authors also found that MANF induced the expression of the neuroprotector HO-1 and protected SH-SY5Y cells from 6-OHDA-induced reactive oxygen species (ROS) [59]. However, this protective effect was completely abolished by the P13K inhibitor LY49002, indicating the involvement of P13K signaling in MANF’s neuroprotective actions. Inclined to this, MANF-treated cells increased the levels of pAkt and GSK3β. Importantly, LY49002 was also shown to suppress MANF-mediated expression of Nrf2 and HO-1. Overall, their study indicated that MANF induces Nrf2 activation through P13K/Akt/GSK3β signaling for its neuroprotective effect [59].

Li et al. found that MANF inhibits apoptosis by reducing caspase 3 levels in rat SAH models, while the Akt inhibitor MK2206, although it did not alter MANF expression, reversed the anti-apoptotic effects of MANF [54]. Administration of MANF in this model was also shown to increase the levels of anti-apoptotic bcl2 and p-MDM2 and decrease the levels of apoptotic bax and p53, indicating that MANF elicits anti-apoptotic effects through modulating Akt/MDM2/p53 signaling [54]. MANF was also shown to suppress neuroinflammation resulting from ischemia. In a mouse cerebral ischemia model induced by middle cerebral artery occlusion (MCAO), supplementation of MANF downregulated the proinflammatory cytokines IL-6, IL-1β, and TNF-α, indicating suppression of neuroinflammation [60].

Superoxide dismutase (SOD) and Glutathione (GSH) are endogenous antioxidants that contribute to cellular detoxification, while Malondialdehyde (MDA) is a product of lipid peroxidation, and its levels indirectly reflect the extent of cell damage. MANF treatment was shown to increase SOD activity and GSH production and subsequently decreased MDA production in MPTP-induced mouse PD model [30]. In addition, pre-treatment with MANF was shown to reduce bax levels and increase the levels of the anti-apoptotic protein bcl2 in SH-SY5Y cells, suggesting that MANF-mediated protection against apoptosis may also involve antioxidant mechanisms [30].

**Figure 3:**
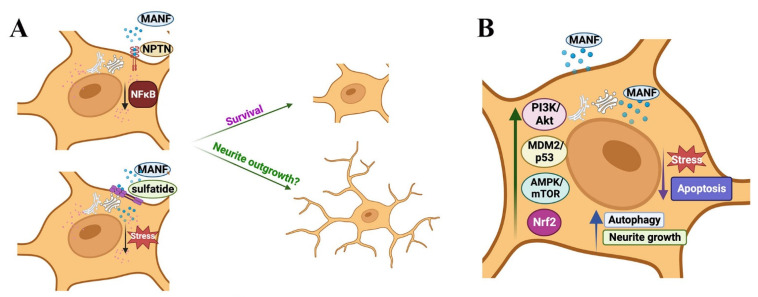
The potential and established mechanisms for MANF’s actions independent of ER stress-UPR axis: (**A**) Extracellular MANF binds to the cell surface: extracellular MANF binds to the lipid sulfatide on the cell surface for cellular entry. MANF also binds to Neuroplastin (NPTN) receptor on the cell surface. MANF’s binding to sulfatide and NPTN and associated signaling promotes cellular survival. Whether MANF promotes neurite outgrowth in a sulfatide or NPTN-dependent manner is unknown. (**B**) MANF promotes cell survival independent of UPR signaling: MANF modulates PI3K/Akt signaling, MDM2/p53 axis, AMPK/mTOR axis, and Nrf2 signaling for its survival, anti-apoptotic, autophagy, and neurite outgrowth promoting actions.

While the studies mentioned above examined the role of MANF in cellular apoptosis, Zhang et al. studied whether MANF contributes to autophagy [46]. In their experiments, they depleted autophagy-related genes in *C. elegans* PD model and found that at least 26 autophagy-related genes may contribute to MANF signaling; they noted that MANF lost its protective effect after the depletion of these genes. The authors of the study suggested that MANF may induce autophagy by modulating the AMPK/mTOR pathway. They also found that the levels of α-synuclein, a promoter of PD, was reduced after induction of autophagy and suggested that MANF may facilitate the clearance of α-synuclein by inducing autophagy [46]. Another recent study demonstrated the role of MANF in chaperone-mediated autophagy (CMA). During CMA, cytosolic proteins that contain a KFERQ motif are recognized by the heat shock protein 70 (Hsc70) and is transported to the lysosomal-associated membrane protein 2A (LAMP-2A), which then carries them to the lysosomal lumen for degradation. MANF was shown to induce the expression of LAMP-2A and Hsc70 in SH-SY5Y cells, indicating that MANF induces CMA. The authors of the study also found that MANF-mediated degradation of α-synuclein is reversed by LAMP-2A siRNA, indicating that MANF-mediated clearance of α-synuclein is CMA dependent. Substantiating this argument further, the authors demonstrated that MANF treatment induces the expression of autophagy-related molecules beclin 1 and LC3 in SH-SY5Y cells in response to α-synuclein challenge. The transcription factor Nrf2 promotes autophagy, and MANF was shown to induce Nrf2 levels in SH-SY5Y cells challenged with α-synuclein, indicating that MANF-dependent autophagy and clearance of α-synuclein may also be mediated by Nrf2. Further, the authors showed that the Nrf2 inhibitor ML385 reversed MANF-induced autophagy. Overall, these observations suggest that the MANF-Nrf2 axis may also be a potential therapeutic intervention point for α-synuclein-driven neurodegenerative disorders, including PD [61]. The already established and potential mechanisms for MANF, independent of ER stress-UPR axis, are illustrated in Figure 3.

## 8. Conclusions and Perspectives

Mounting evidence indicates that MANF offers cellular protection in PD, AD, and stroke. While these pre-clinical results are encouraging, whether MANF supplementation is a clinically viable and efficacious approach to managing the above-mentioned disorders needs additional investigation. In addition to the above-mentioned disorders, the knowledge of MANF signaling in other neurological conditions is also emerging. Multiple sclerosis is an autoimmune disorder. Inflammatory lesions and demyelinating plaques are the most common pathological hallmarks of MS [62]. Dexamethasone is an immunosuppressant commonly used for delaying the progression of MS. Dexamethasone treatment was shown to upregulate MANF in the lumbar spinal cord of a mouse model of experimental autoimmune encephalomyelitis (EAE). Interestingly, EAE mice supplemented with hMANF demonstrated better locomotor functions compared to controls, suggesting that MANF may protect motor neurons in MS [63]. Similarly, epilepsy is a chronic condition characterized by unprovoked seizures. It affects almost 50 million people worldwide. Lindholm et al. showed that MANF expression is upregulated in the dentate granule cell layer, piriform and parietal cortex, and the thalamic reticular nucleus of epileptic mice, suggesting a potential functional role for MANF in epilepsy [64].

Fundamental studies have demonstrated the ability of MANF to modulate ER stress-UPR axis, PI3K/Akt signaling, MDM2/p53 axis, AMPK/mTOR axis, and Nrf2 signaling for its cell survival, anti-apoptotic, antioxidant, and autophagy properties. Several studies also showed MANF’s ability to induce neurite outgrowth. For example, MANF was shown to promote neurite outgrowth in DA neurons and N2a cells [2,65]. Additionally, MANF was shown to be essential for neurite extension in the developing mouse cortex. For instance, Tseng et al. showed that MANF is expressed in neural crest cells and its deficiency leads to inhibition of neuron differentiation and outgrowth [66]. While these studies also attribute MANF’s ability to modulate ER stress-UPR axis and Akt signaling for its neurite-promoting effect, the exact receptor involvement underlying its neurotrophic actions is still unknown. It is now clear from the past studies that MANF binds to GRP78 for potentially modulating UPR signaling and binds to sulfatide on the cell surface for cellular uptake. However, whether MANF binding to sulfatide and GRP78 has any direct influence on facilitating neurite outgrowth is not well understood. A recent study by Yagi et al. demonstrated that MANF binds to the neuroplastin (NPTN) receptor on the cell surface, and the MANF-NPTN axis was shown to inhibit NFκB signaling and cell death [67]. However, whether MANF-NPTN axis promotes neurite outgrowth is unknown.

Currently, not much is known about the role of MANF in the peripheral nervous system (PNS), especially its role in peripheral nerve injury and repair. Intrinsic regeneration of peripheral nerves does not sustain for longer periods due to insufficient availability of growth factors in the regenerative milieu, and this situation is often compounded by the expression of growth suppressors or tumor suppressor class of proteins in the milieu [58,68]. Therefore, functional recovery after peripheral nerve injury is often incomplete. In addition, no pharmacological therapies are effective in repairing injured peripheral nerves. Our recent work demonstrated that in vitro growth primed dorsal root ganglia (DRG) is an ideal model for exploring molecular targets for peripheral nerve regeneration [69]. A comparative proteomics study employing both in vitro and in vivo growth-primed DRGs revealed that MANF may be a potential molecular candidate for promoting peripheral nerve repair. We also found that MANF is expressed predominantly in NF200^low^ sensory neurons, and demonstrated that MANF induces neurite outgrowth in peripheral neurons in vitro, further suggesting that MANF may be a potential therapeutic candidate for peripheral nerve repair [70]. However, additional in vivo studies are warranted for making conclusive remarks. Similarly, the potential role of MANF in peripheral neuropathies such as diabetic neuropathy, chemotherapy-induced peripheral neuropathy, and autoimmune disease-related peripheral neuropathies is an open area for investigation. Given the detrimental roles of aberrant UPR signaling in painful neuropathies, it is highly likely that MANF may be protective in these scenarios. Similarly, MANF signaling in other neurodegenerative conditions, including spinal cord injury is not well-understood. Although MANF was initially discovered as a secretory protein of glial cell origin, the neurons also synthesize MANF. A potent growth factor neuregulin was also shown to have a similar dual source origin for its seamless availability for natural nerve repair [71]. Whether MANF is indispensable for neuron repair needs additional investigation. Overall, detailed in vivo studies are warranted to reveal the therapeutic utility of MANF in additional neurodegenerative disorders, including PNS disorders.

## Figures and Tables

**Table 1:** Actions of MANF in PD, AD, and stroke.

MANF in PD	MANF in AD	MANF in STROKE
MANF promotes the survival of DA neurons [2]. MANF elicits long-term neuroprotective effects on the nigrostriatal DA system in rat PD models [48]. PD patients express increased levels of MANF [50].	MANF protects neurons from Aβ induced toxicity [25]. High number of MANF-positive neurons are found in the brain samples of AD patients compared to non-AD samples [51]. MANF deficiency accentuates neurotoxicity, while its supplementation reduces neurotoxicity [25].	MANF levels are upregulated in subarachnoid haemorrhage (SAH) stroke models [54]. MANF facilitates functional improvement after stroke [55]. MANF increases blood flow to the brain in rat stroke models [57]. MANF upregulates the expression of angiogenic markers CD34, VEGF, Ang1 in the peri-infarct cerebral cortex, contributing to angiogenesis [57].
